# Enabling Scalable Nanoscale 3D Printing via Laser‐Driven Termination

**DOI:** 10.1002/smll.202514504

**Published:** 2026-08-04

**Authors:** Chanbin Yoo, Jongcheon Bae, Jung Woo Lee, Ji‐Tae Hong, Jun Chang Yang, Seung Kwon Seol, Jaeyeon Pyo

**Affiliations:** ^1^ Smart 3D Printing Research Team Korea Electrotechnology Research Institute (KERI) Changwon South Korea; ^2^ Electric Energy & Materials Engineering KERI School University of Science and Technology (UST) Changwon South Korea; ^3^ School of Materials Science and Engineering Pusan National University Busan South Korea

**Keywords:** 3D printing, laser ablation, meniscus writing, nanostructure, polymer

## Abstract

Nanoscale three‐dimensional (3D) printing promises to revolutionize next‐generation devices in optics and electronics, but its widespread adoption is hindered by fundamental challenges in scalability and manufacturing throughput. Meniscus‐guided writing is a promising technology for fabricating 3D nanostructures, yet it suffers from a critical limitation in its termination mechanism. The conventional rapid‐retraction method is effective only for low‐viscosity, low‐concentration inks, severely restricting printing speed and material selection. Here we overcome the bottleneck by developing a laser‐driven termination method. We integrated a 405 nm focused laser co‐axially with a side‐view optical microscopy monitoring system to induce on‐demand thermal ablation, cleanly severing the printed structures. By replacing the concentration‐sensitive physical separation with a thermal process, our method decouples termination from ink rheology, a breakthrough enabling high‐concentration ink (> 1.6 mg/mL) usage, unlocking printing speeds up to 1000 µm/s—a 100‐fold throughput increase compared to the retraction‐based process. The laser‐driven approach also provides exceptional geometric freedom. We demonstrate precise, on‐demand height control and the fabrication of freestanding tilted structures. The method's versatility was confirmed for a range of sizes, material systems, and substrates. Our approach provides a robust and scalable platform for the omnidirectional manufacturing of complex 3D nanostructures.

## Graphical abstract

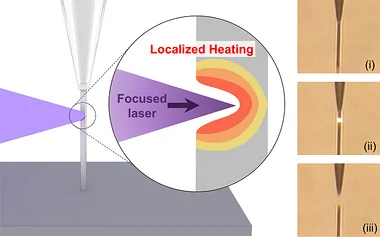

## 1 Introduction

Nanostructures provide high‐resolution integration and unique electronic and optical properties originating from their morphological characteristics [1, 2, 3]. Polymer nanostructures represent a promising material system owing to cost‐effectiveness and solution processibility. A combination of low cost and solution processibility makes polymer nanostructures highly suitable for applications across various disciplines, including optics [4, 5], electronics [6, 7], and optoelectronics [8, 9]. The wide range of applications for polymer nanostructures requires versatile fabrication methods capable of tailoring polymers with desired dimensions to control morphological properties. Representative fabrication methods include self‐assembly [10, 11], lithography [12, 13], and three‐dimensional (3D) printing [14, 15]. 3D printing is particularly promising, as it enables the fabrication of three‐dimensional nanostructures through simple and low‐cost processes. Over a decade ago, a nanoscale 3D printing method termed meniscus‐guided writing was developed, a technique that fabricates 3D nanostructures by guiding a femtoliter liquid meniscus for solidification [16]. The technique has since been widely adopted for fabricating 3D nanostructures in diverse fields [5, 14, 15, 16, 17, 18, 19, 20, 21, 22, 23, 24, 25, 26, 27, 28, 29, 30, 31, 32, 33, 34, 35, 36, 37, 38, 39, 40, 41, 42, 43, 44, 45, 46, 47]. The technique operates by forming a femtoliter‐scale polymer ink meniscus between a nanoscale nozzle and a substrate. Rapid solvent evaporation from the meniscus during its guided movement induces ink solidification, directly forming the polymer nanostructure.

Despite its versatility, meniscus‐guided writing is constrained by its termination mechanism, which limits usable ink concentrations. A conventional termination strategy involves rapid nozzle retraction to induce meniscus separation [14, 15]. The key factor for meniscus separation is the relationship between meniscus stability and retraction speed; sufficiently rapid retraction is required to induce the separation of the meniscus from the solidified structure, thereby terminating subsequent fabrication. In practice, termination by rapid retraction is restricted to low‐viscosity, low‐concentration inks. The use of low‐viscosity, low‐concentration inks impedes the enhancement of printing speeds due to the prolonged time required for solidification, consequently limiting improvements in productivity.

To overcome these rheological constraints, a termination mechanism operating independently of the ink's characteristics is required. Here, we propose an active termination strategy that concludes the process by precisely ablating the solidified polymer filament to separate the printed structure from the active liquid meniscus. For the local manipulation of printed polymer nanostructures, high‐resolution tools such as scanning probe lithography (SPL) [48, 49], thermal scanning probe lithography (t‐SPL) [50, 51], focused ion beam (FIB) [52, 53], and laser [54, 55, 56] have been utilized. However, contact‐based methods such as SPL and t‐SPL are difficult to integrate into the in situ printing process due to spatial constraints and the risk of physical interference with the nozzle. Meanwhile, FIB requires a high‐vacuum environment, leading to complex equipment setups and substantial operational costs. In contrast, laser‐based fabrication is performed in an ambient atmosphere and in a non‐contact mode, offering the advantage of rapid processing without physical interference or complex setups.

Various laser‐based fabrication technologies have been utilized across diverse disciplines, as summarized in Figure S1. However, there remain difficult aspects to achieving nanoscale 3D polymer fabrication using each technique. Laser ablation is effective for planar surface patterning by partially cutting structures with a laser [54, 55, 56]. However, since the laser direction is fixed perpendicular to the substrate, it is unsuitable for fabricating complex 3D structures and can cause thermal damage to the underlying substrate. Similarly, laser direct writing technology, which directly applies a reactive layer for localized curing or self‐assembly, enables rapid fabrication but faces difficulties in 3D structural fabrication and risks thermal diffusion into underlying layers [57, 58]. While Laser‐induced forward transfer (LIFT) deposits material by melting it from a donor film with a laser and depositing it onto a target substrate, its droplet‐based transfer mechanism makes it difficult to form continuous and smooth nanostructures [59, 60, 61]. Two‐photon printing can fabricate structures with high resolution through partial polymerization in resin where energy is concentrated via localized photon density by locally focusing the laser, but it requires specialized photosensitive materials to trigger the two‐photon excitation [62, 63]. Furthermore, while hybrid technologies combining laser with direct ink writing can produce high‐performance structures by manipulating material properties simultaneously with printing using a laser, they often struggle to fabricate nanoscale 3D structures due to the resolution of these technologies [64, 65].

Here, we present a versatile 3D printing strategy for fabricating nanoscale 3D polymer structures using a laser‐driven termination method, which enables damage‐free processing on various substrates. Unlike laser‐based methods that directly apply the substrate vertically and risk thermal damage to surrounding areas, our technique integrates a side‐illumination laser with meniscus writing to focus the energy on the printed polymer structure. The laser beam is tightly focused to generate localized heating on the printed polymer structure, inducing ablation without affecting adjacent structures. Such localized ablation prevents thermal diffusion into the substrate and previously fabricated structures, thereby ensuring process stability for integrated devices and underlying materials.

Notably, the method is applicable to high‐concentration inks, for which termination by rapid retraction was not feasible. Employing high‐concentration inks allows stable meniscus formation at higher printing speeds, a pivotal capability expected to significantly improve the throughput. We confirmed the versatility of the method through application to various nozzle diameters, polymer materials, and substrate materials. We anticipate the approach will be particularly useful in optics and electronics, where the fabrication of dense nanostructure arrays is often required. The laser‐driven termination enables the use of high‐concentration polymer inks, bypassing the speed limitations inherent in retraction‐based methods. The resulting high throughput makes the large‐scale fabrication of such nanostructure arrays feasible, directly addressing a manufacturing bottleneck for advanced devices.

## 2 Laser‐Assisted Nanoscale 3D Printing

Figure 1a illustrates the nanoscale 3D printing method combined with focused laser ablation. The method utilizes meniscus writing, employing solvated polymer inks and nanoscale nozzles, to fabricate nanoscale polymer structures. A detailed description of the meniscus writing process is provided in Figure S2. Unlike the conventional meniscus writing method, termination is achieved by localized laser ablation. Compared to the previous method of rapid retraction, the termination process is precisely controlled by accurately positioning the localized heating, which is induced by a focused 405 nm laser beam.

**FIGURE 1:**
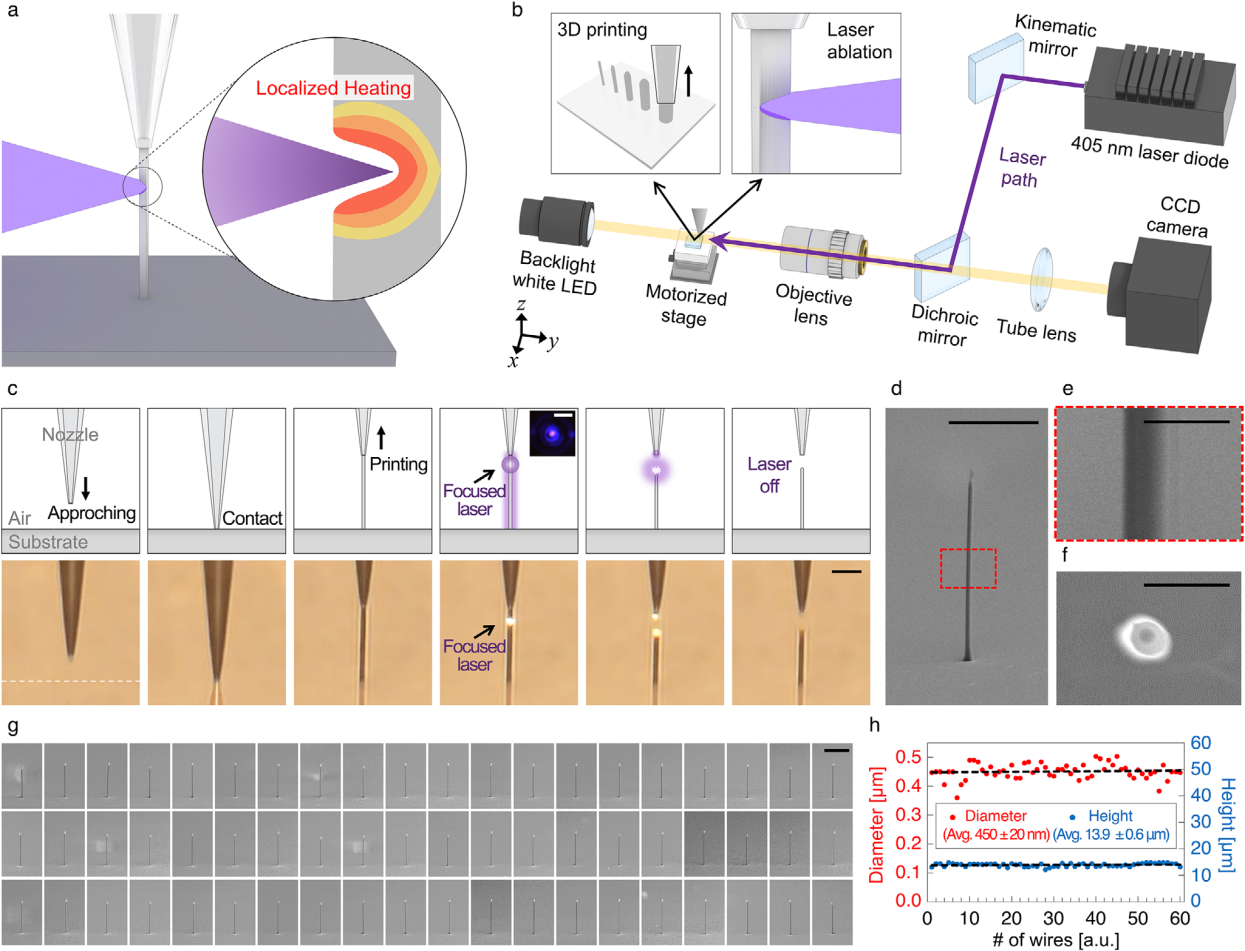
System and process for laser‐driven nanoscale 3D printing. (a) Conceptual illustration of polymer nanostructure printing combined with laser ablation. (b) Schematic of the custom‐built experimental setup integrating the laser module and co‐axial optical microscopy monitoring for 3D printing and laser ablation. (c) Schematic illustrations and corresponding optical microscopy (OM) images of the fabrication procedure, filtered by a notch filter (405 nm). Inset: OM image of the focused laser spot. Scale bars: 10 µm. Inset scale bar: 3 µm. (d) Side‐view SEM image of fabricated polymer nanostructures. Scale bar: 10 µm. (e,f) Magnified SEM image from (e) side view and (f) top view. Scale bars: 1 µm. (g) SEM images of 60 nanowires fabricated in series. Scale bar, 10 µm. (h) Diameters and heights of the printed nanowires.

A custom‐built experimental system (Figure 1b) performs the 3D printing and laser ablation simultaneously. The system consists of three components: a laser source module, a high‐resolution optical microscope (OM), and a precision motion system. The high‐resolution OM module (composed of an infinity‐corrected objective lens, a tube lens, and a charge‐coupled‐device (CCD) camera) provides detailed and accurate observation of the printing process in real‐time. The laser module is designed to launch a focused laser beam onto the printing region using the objective lens through a kinematic mirror and a dichroic mirror. The co‐axial alignment of the OM and the laser module is critical, as it permits the operator to precisely target the laser beam onto the nanostructure through the real‐time optical image. Accurate placement of the nanoscale nozzle and target substrates, with a 100 nm step resolution, is achieved using motorized stages controlled by custom‐developed software. Detailed information on each component is provided in Section 7.

The typical procedure for 3D printing a polymer nanostructure is illustrated in Figure 1c with schematics and corresponding in situ optical images. First, a nanoscale nozzle filled with a polymer ink is positioned near the desired location on the target substrate. Second, a femtoliter liquid meniscus is formed on the substrate upon contact between the aperture of the ink‐filled nanoscale nozzle and the substrate surface. Third, as the nozzle moves upward, a freestanding nanoscale structure is drawn from the meniscus, solidifying rapidly due to solvent evaporation. Finally, the printing process is terminated by focusing a laser beam onto the desired position of the printed structure for localized ablation. The fabricated polymer nanostructure was then observed using scanning electron microscopy (SEM) in side view (Figure 1d,e) and top view (Figure 1f). The fabricated polymer nanostructure exhibits a vertically freestanding nanowire structure with a smooth surface and a circular shape. We printed 60 nanostructures arranged in a nanowire array at once to estimate the repeatability of the printing process, as shown in Figure S3. The geometrical characteristics of the printed nanowires were then investigated using SEM (Figure 1g). The repeatability of the printing process for a given nozzle was estimated, averaging 450 nm for the diameter and 13.9 µm for the height, with standard deviations of 20 nm (4.4%) and 0.6 µm (4.3%), respectively (Figure 1h). The resulting arrays of nanowires exhibit uniform diameters and heights, validating the high reproducibility of the laser‐driven nanoscale 3D printing process.

## 3 High‐Throughput Printing via Rheology Decoupling

We compared the two termination methods—conventional rapid retraction (Figure 2a) and laser ablation (Figure 2b)—using various ink formulations to investigate the effect of ink concentration on the termination process. Detailed information on these two termination methods is provided in Figure S4. The polymer inks were prepared by dissolving polystyrene powder in o‐xylene at various concentrations from 0 to 20 mg/mL. Rheological properties of the inks are provided in Figure S5. While the 20 mg/mL ink exhibits notably higher viscosity than others, all inks exhibit Newtonian behavior similar to pure o‐xylene solvent. It is noteworthy that, unlike extrusion‐based direct ink writing (DIW), the meniscus writing method relies on solvent evaporation rather than shear thinning.

**FIGURE 2:**
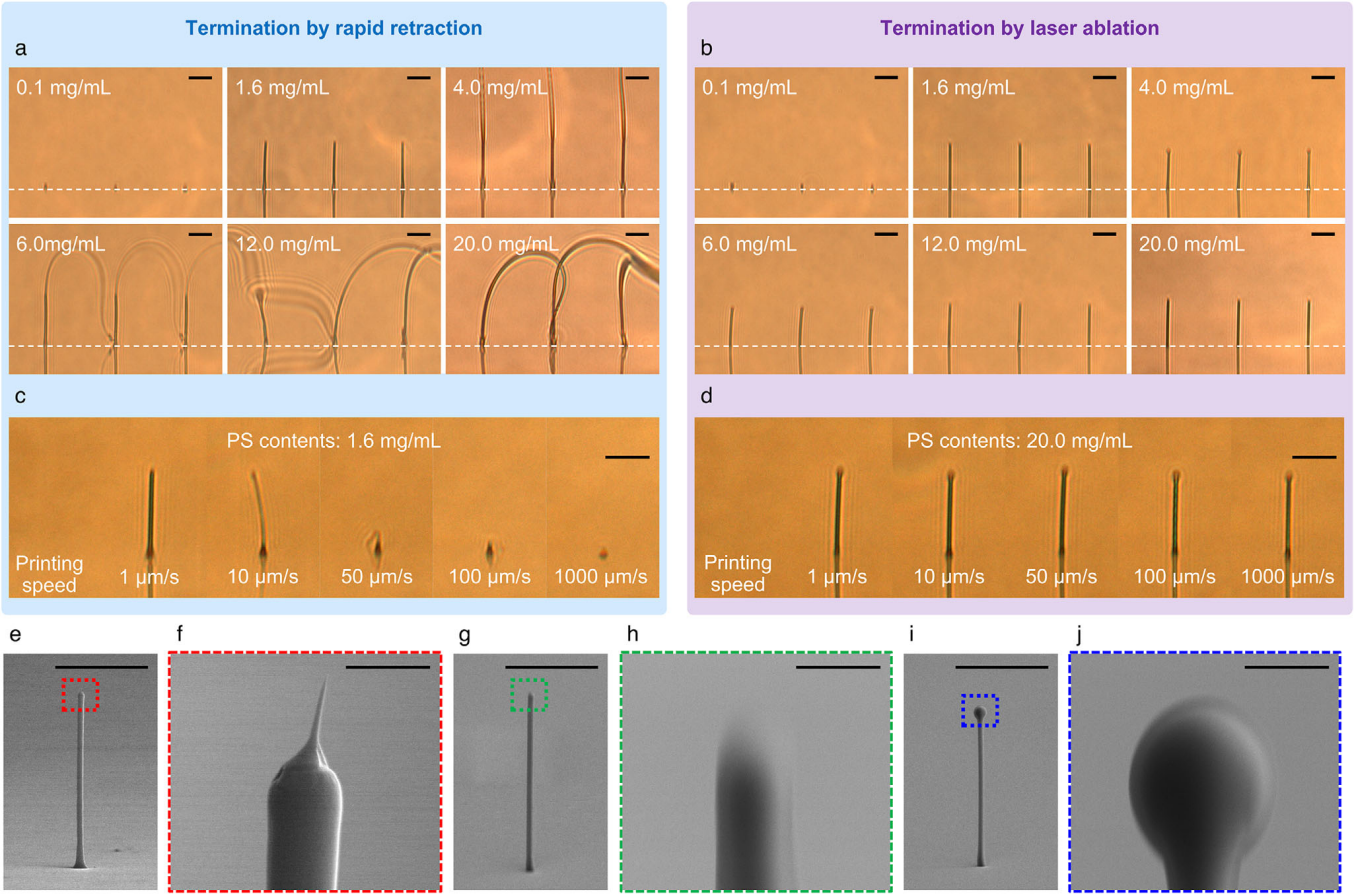
Comparison of termination methods and ink formulations. (a,b) OM images of nanowires fabricated with polymer inks of varying concentrations terminated by (a) the rapid‐retraction method and (b) the laser ablation method. Scale bars: 10 µm. (c,d) OM images comparing fabrication stability at increasing printing speeds (1–1000 µm/s) for (c) the rapid‐retraction method (1.6 mg/mL) and (d) the laser ablation method (20 mg/mL). Scale bars: 10 µm. (e–j) SEM images of nanowires fabricated using different termination methods. (e,g,i) Side‐view SEM images of representative nanowires fabricated by (e) rapid retraction and by laser ablation under (g) optimized and (i) overexposed laser conditions. (f,h,j) Magnified SEM images of the nanowire ends after termination. Scale bars: 10 µm (e,g,i), 1 µm (f,h,j).

During nanoscale 3D printing, the meniscus ink develops rheological properties distinct from the bulk ink inside the nozzle reservoir. The femtoliter‐scale meniscus possesses a high surface‐to‐volume ratio, causing rapid solvent evaporation. As the solvent evaporates, the polymer concentration increases within the meniscus. Consequently, the ink transitions from its original dilute Newtonian state to a highly concentrated viscous state, enabling the formation of solid polymer structures.

Termination by rapid retraction is governed by a competitive interplay between two speed‐dependent processes: solidification (driven by solvent evaporation) and nozzle movement. Printing occurs when the nozzle movement is slow enough for evaporation to sufficiently increase the polymer concentration within the meniscus, inducing solidification. Termination, conversely, occurs when the nozzle moves sufficiently rapidly to rupture the meniscus before the polymer concentration reaches the solidification threshold. Therefore, low bulk ink concentration is a prerequisite for the competitive mechanism to function. The low concentration maintains the meniscus in a low‐viscosity state, permitting rapid retraction termination to complete before solidification.

A fundamental physical contradiction arises from this mechanism. To achieve retraction‐based termination, the bulk ink concentration must be low. However, to achieve high‐speed printing (and thus high productivity), the bulk concentration must be high to accelerate solidification. The rapid retraction method therefore imposes a narrow processing window (Figure 2a). In practice, retraction‐based termination was effective only at 1.6 mg/mL. At lower concentrations (e.g., 0.1 mg/mL), the meniscus failed to stabilize for printing. At concentrations above 1.6 mg/mL, the ink became too viscous for rapid retraction to work, resulting in termination artifacts (Figure 2a). Consequently, the conventional termination method restricts the printing speed, which in turn severely limits manufacturing productivity.

Laser‐driven termination overcomes the aforementioned speed restriction by decoupling the termination process from the constraint of ink concentration, facilitated by direct thermal ablation. Because termination is no longer reliant on a low‐viscosity state, the bulk ink concentration can be set much higher. Our experimental results confirm the processability at higher‐concentration inks, exceeding 1.6 mg/mL (Figure 2b). Laser‐driven termination succeeded across all tested concentrations (1.6–20 mg/mL), effectively removing the upper concentration boundary that restricted productivity in the conventional process.

The processability of high‐concentration inks (>1.6 mg/mL) is the key to unlocking high‐throughput fabrication. Higher concentration inks enable faster solidification, which permits correspondingly faster printing speeds without fabrication failure. Figure 2c,d provide a direct quantitative experimental comparison. The conventional retraction method is inherently confined to 1.6 mg/mL concentration, which limits printing speeds below 10 µm/s for stable fabrication (Figure 2c). In contrast, the laser‐driven method accommodates 20 mg/mL concentration inks, unlocking printing speeds up to 1000 µm/s, effectively an increase of two orders of magnitude in throughput (Figure 2d). The laser‐driven approach thus provides a pivotal key to establishing nanoscale 3D printing as a scalable manufacturing technology.

Furthermore, the thermal ablation mechanism cleanly severs the polymer structure, preventing the filamentary defects associated with the incomplete physical separation of rapid retraction as shown in Figure 2a. The different mechanisms also impart distinct tip morphologies: physical separation (retraction) stretches the meniscus, resulting in a tapered filamentary end (Figure 2e,f), while thermal ablation (laser) melts the polymer, allowing surface tension to form a smooth end (Figure 2g,h). Notably, if the laser continues to apply the structure after termination, it transfers additional thermal energy, forming a spherical end (Figure 2i,j). This phenomenon indicates that the terminal spherical shape can be effectively controlled by adjusting the laser exposure time. Such morphological characteristics, tunable through laser exposure time, offer a promising option for diverse applications related to the optical properties of the spherical structures.

The robustness of the laser‐based approach also addresses the practical challenge of intermittent printing, which is often required during complex fabrication. Pauses during the printing process cause solvent evaporation at the nozzle tip, increasing the local ink concentration. The conventional retraction method, being highly sensitive to concentration, fails under such conditions; the increased viscosity impedes clean separation, resulting in filamentary defects upon termination (Figure S6a). In contrast, the laser‐driven approach remains unaffected by the evaporation‐induced concentration shift, cleanly severing the material even after the prolonged delay (Figure S6b). The demonstrated robustness is important for practical manufacturing, allowing complex fabrication that involves intermittent steps.

## 4 Geometric Control

Individual height control is a key advantage of 3D printing, facilitating material positioning in three‐dimensional space at will, a capability often required for advanced applications in displays, electrodes, sensors, data storage, anticounterfeiting, and others. We demonstrate 3D height control via laser‐driven ablation in two distinct strategies: in situ (during printing) and post‐processing (after printing).

The in situ strategy (Figure 3a) integrates the termination process directly into the printing sequence for real‐time height control. The focused laser is positioned in proximity to the stationary nozzle tip (3 µm below). Printing occurs by moving the substrate relative to the fixed nozzle tip, with the laser spot positioned at the same offset. As the printed structure reaches the desired height, the laser beam is activated to sever the structure and then turned off for the subsequent printing process (Figure 3b). The in situ method enables agile, real‐time height control using a simple, integrated system. We demonstrated the in situ strategy by fabricating six nanostructures with varying heights in Figure 3c.

**FIGURE 3:**
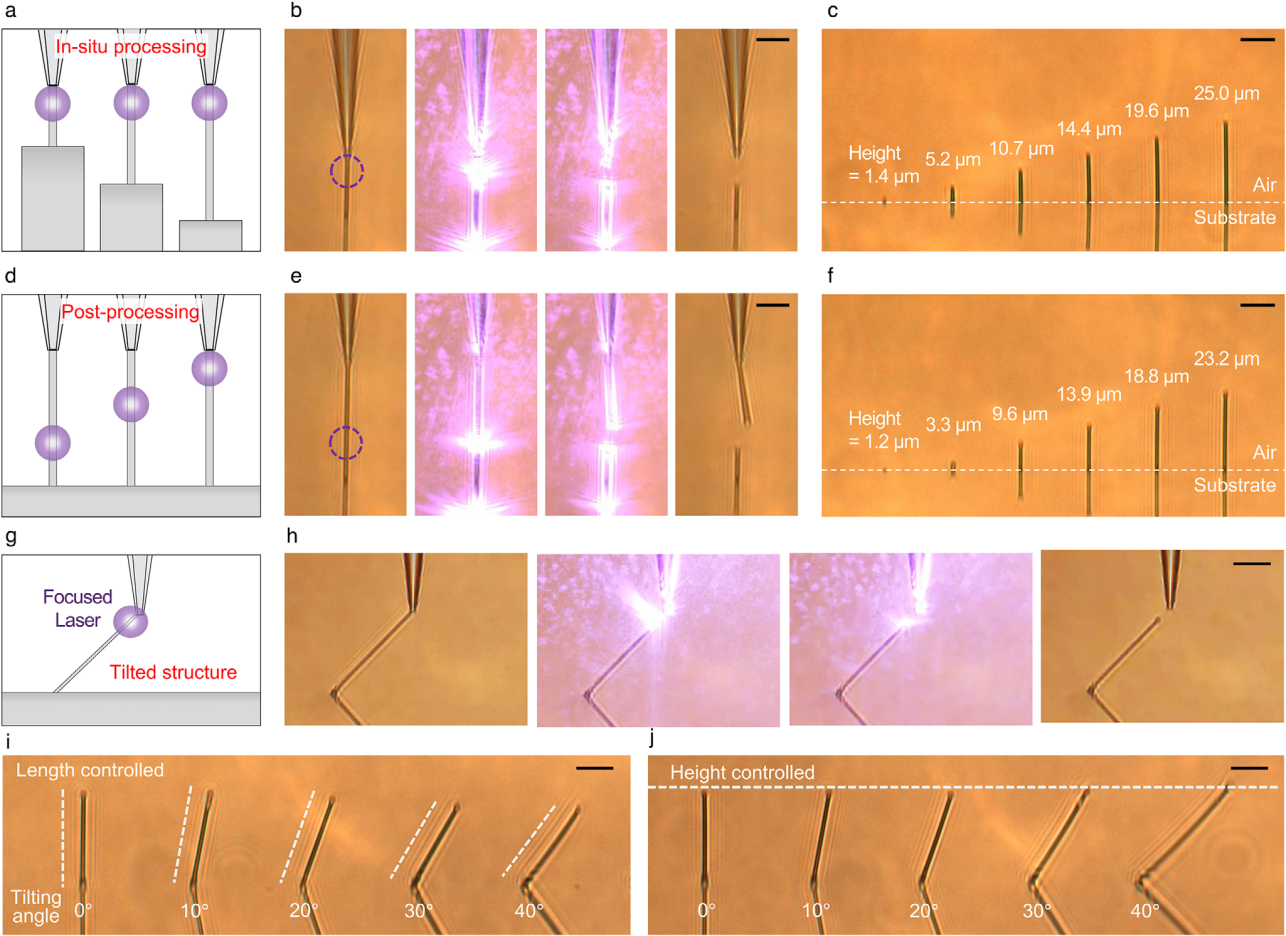
Geometric control of polymer nanowires using laser termination. (a–f) Fabrication of vertical nanowires with controlled heights using (a–c) in situ processing and (d–f) post‐processing strategies. (a,d) Schematic illustrations. (b,e) OM images showing the fabrication procedure. (c,f) OM images of the resulting printed vertical nanowires. (g–j) Fabrication of freestanding tilted structures. (g) Schematic illustrations for terminating tilted structures. (h) OM images showing the procedure. (i,j) OM images of the resulting tilted nanowires, demonstrating (i) controlled printed length (via in situ) and (j) controlled vertical height (via post‐processing). Scale bars: 10 µm.

The post‐processing strategy (Figure 3d) involves termination as a separate step after the printing is completed, where the laser beam is used as a high‐precision trimming tool. The focused laser is applied at the target position on the printed structure to sever at the desired height (Figure 3e). Notably, after trimming, the remaining nanostructure dangling from the nozzle tip is drawn back into the nozzle, where it dissolves, allowing subsequent printing. We fabricated nanowires with controlled height via the post‐processing strategy (Figure 3f). The post‐processing strategy is ideal for applications requiring uniform height definitions or precise length trimming, as it eliminates the need for time synchronization with the printing process. The strategy is also readily scalable to multiple nozzle systems, enabling large‐area and rapid fabrication. A comparison of Figure 3c,f confirms that both methods produce nanoscale structures of controlled height.

The laser‐driven height control is also applicable to non‐vertical (tilted) structures (Figure 3g,h). Fabricating freestanding tilted structures is challenging for the conventional retraction method. The difficulty arises because as the printing trajectory tilts, the linear alignment between the nozzle tip and the wire is lost, creating an angled joint spanned by the meniscus bridge. The resulting asymmetric force profile impedes a clean physical separation. In contrast, the laser‐driven method remains unaffected by the printing angle, enabling precise termination of tilted structures.

We confirmed the termination capability on tilted geometries by fabricating nanostructures at tilt angles from vertical to 40°. Structures tilted at angles greater than 40° were also fabricated (Figure S7), although they show bending toward the substrate due to the limited mechanical modulus of the polystyrene nanowires. We applied both strategies to these tilted structures: the in situ strategy is ideal for controlling the path length (Figure 3i), while the post‐processing strategy allows for precise trimming at a uniform vertical height (Figure 3j).

The demonstrated results confirm that the laser‐driven approach overcomes fundamental geometric constraints of conventional meniscus writing. The development of in situ and post‐processing strategies provides precise, on‐demand height control, while the angle‐independent termination introduces a new degree of fabrication freedom. The method thus offers a robust pathway for fabricating complex, freestanding nanostructures with controlled geometries and orientations, enabling scalable omnidirectional nanoprinting.

## 5 Process Generality

3D structures are used in applications spanning the nanometer to the micrometer scale. A versatile fabrication method should therefore be applicable to a wide range of structure sizes. We evaluated the versatility of the laser‐driven termination using nozzles with tip diameters of 0.6, 1.0, 4.0, and 8.0 µm, which determines the final diameter of the printed structures (Figure S8). In situ OM imaging (Figure 4a) confirms successful termination for all nozzle diameters. The time required for complete ablation increases with the nozzle diameter (Figure 4b), a trend attributed to the larger polymer volume requiring greater laser energy input. The predictable scaling of ablation time with diameter confirms the process is robust and controllable, enabling its application across both nanometer and micrometer scales.

**FIGURE 4:**
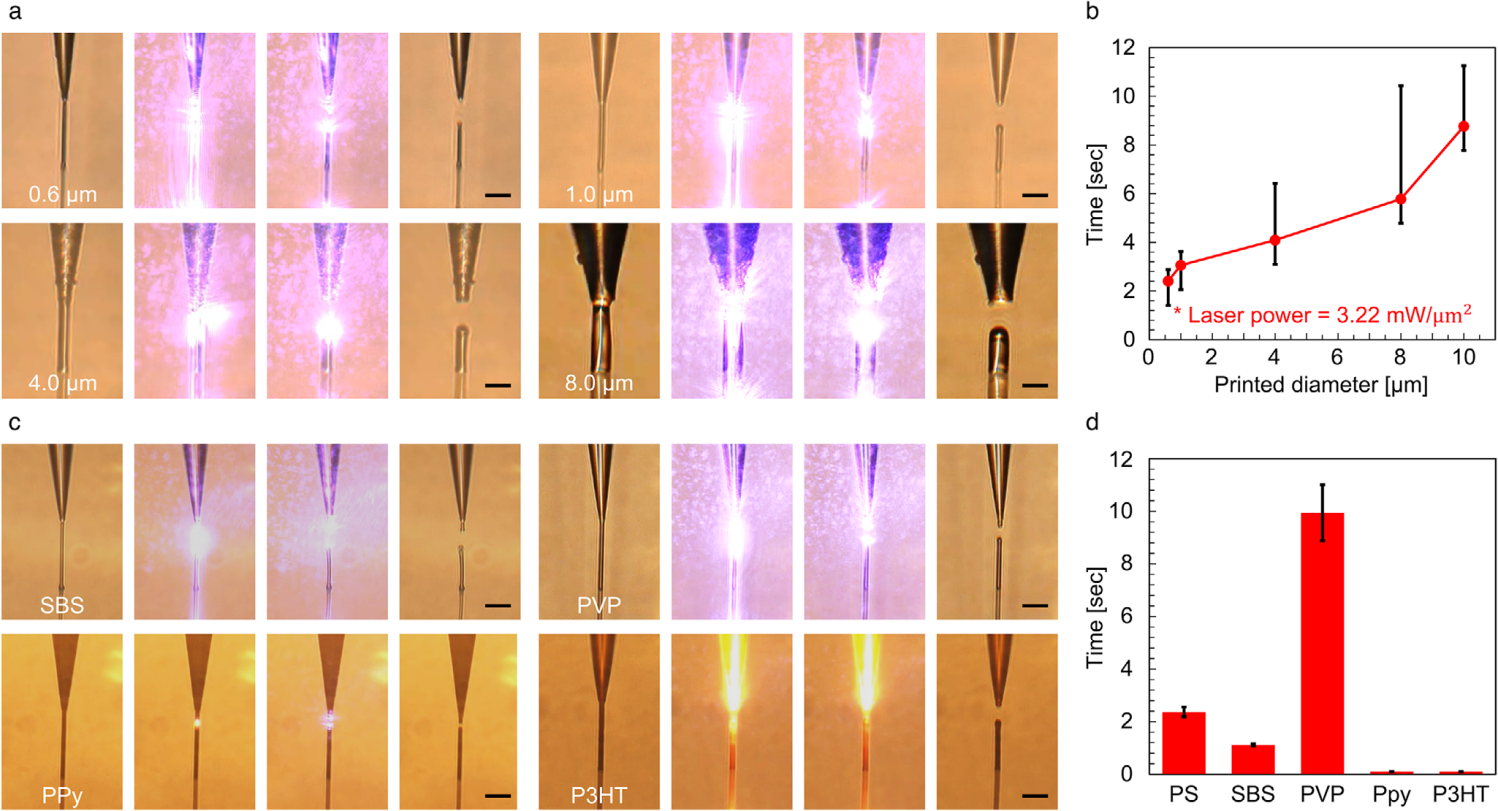
Process generality across scales and materials. (a) Laser termination of polymer structures printed from nozzles with varying inner diameters: 0.6, 1.0, 4.0, and 8.0 µm. (b) Laser ablation time as a function of nozzle inner diameters. (c) Laser termination demonstrated for diverse polymer systems: poly(styrene‐block‐butadiene‐styrene) (SBS), Polyvinylpyrrolidone (PVP), polypyrrole, and Poly(3‐hexylthiophene‐2,5‐diyl) (P3HT). (d) Laser ablation time measured for each polymer system. Scale bars: 10 µm.

Given the widespread application of 3D structures, a general fabrication method should be compatible with various polymers and solvents. We further confirmed the material generality of the method beyond the demonstrated polystyrene, testing diverse polymers: Poly(styrene‐butadiene‐styrene) (SBS), Polyvinylpyrrolidone (PVP), Polypyrrole, and poly‐3‐hexylthiophene‐2,5‐diyl (P3HT) (Figure 4c). The inks were prepared using adequate solvents (o‐xylene, water, toluene). Laser ablation operated successfully for all polymers–solvent combinations, confirming the process is versatile across diverse material systems. The success with chemically distinct systems—such as conjugated polymers in non‐polar solvents (P3HT in toluene) and polar polymers in aqueous solutions (PVP in water)—highlights its robustness against varying ink chemistries.

Notably, the opaque materials (Polypyrrole, P3HT) showed markedly reduced laser scattering in the OM images compared to the others (Figure 4c), indicating high absorption of the laser energy. This high absorption resulted in highly efficient termination, as confirmed by their significantly shorter ablation times (Figure 4d). In contrast, transparent materials (SBS, PVP) showed more scattering but were also terminated successfully. The overall differences in ablation time (Figure 4d) are thus attributed to the distinct optical and thermal properties of each polymer.

Finally, we evaluated the method's applicability on diverse substrate materials. Figure S9 shows successful fabrication of laser‐terminated polymer nanowires on silicon (semiconductor), platinum and copper (electrodes), and PET (flexible substrate), confirming the method is substrate‐independent.

The substrate versatility is a key benefit of our side ablation scheme, which is aligned using side‐view OM monitoring. The laser targets the polymer structure laterally, above the substrate, ensuring the termination process is unaffected by the substrate's physical properties. In contrast, conventional top‐view laser ablations would be strongly affected by substrate properties (e.g., thermal conductivity, reflectivity), leading to reflections, unintended substrate damage, or process‐related residues. Our configuration efficiently bypasses these limitations, making it crucial for fabricating 3D structures directly onto sensitive, pre‐patterned electronics or flexible devices without causing damage.

The combined results confirm that the laser‐driven termination can be a general method for various scenarios, effectively overcoming the physical and chemical constraints of structure size, ink formulation, and the underlying substrate.

## 6 Conclusion

In conclusion, we developed a laser‐driven termination method that overcomes the fundamental constraints of conventional nanoscale 3D printing technology of meniscus writing. By replacing the concentration‐sensitive physical retraction with laser‐induced thermal ablation, the termination process is rendered independent of ink viscosity. The advancement is key to realizing high‐throughput manufacturing of nanoscale 3D printing. It enables the use of high‐concentration inks (>1.6 mg/mL), which were previously incompatible with meniscus writing, unlocking printing speeds up to 1000 µm/s—an increase of two orders of magnitude. Furthermore, the laser‐based approach introduces notable geometric freedom. We demonstrated in situ and post‐processing laser ablation strategies for precise, on‐demand height control. The printing‐angle‐independent nature of the laser termination overcomes the asymmetric force profiles, enabling the fabrication of freestanding tilted structures, a key step toward omnidirectional nanoprinting. We confirmed the versatility of the method for a range of sizes, materials, and substrates. The side ablation scheme allows the laser ablation process to be unaffected by the substrate materials. The laser‐driven approach provides a robust, scalable, and omnidirectional printing platform for fabricating complex 3D nanostructures for advanced optics and electronics.

## 7 Methods

### 7.1 Preparation of Printing Nozzles

Printing nozzles were fabricated from borosilicate glass capillaries (inner diameter 0.5 mm, Sutter Instrument, USA). The capillaries were deformed by pulling under localized laser heat using a laser puller (P‐2000, Sutter Instruments, USA), producing tapered microcapillary nozzles suitable for nano‐micro scale printing. The dimensions of the printing nozzles were measured by SEM.

### 7.2 Experimental Setup

The experimental setup of nanoscale printing consisted of precision step motors (Kohzu, Japan) configured as motion modules for nozzle control (X, Y, Z) and substrate positioning (X, Y, Z, A, B), operated by custom‐built software for real‐time manual control and g‐code‐based automated control. Real‐time monitoring of the printing process was performed using an integrated high‐resolution transmission OM system comprising a collimated LED backlight (548 mW, T = 6500 K, spot size = 50 mm, Thorlabs, USA), an infinity‐corrected objective lens (FL = 4.0 mm, NA = 0.55, Mitutoyo, Japan), a tube lens (FL = 200 mm, Mitutoyo, Japan), and a CCD camera (BFS‐U3‐70S7C‐C, Teledyne FLIR, USA). The laser module comprised a 405 nm GaN diode laser (MLL‐III‐405‐200 mW, Changchun New Industries Optoelectronics Technology Co. Ltd, China) and a power supply (PSU‐III‐FDA, Changchun New Industries Co. Ltd, China).

### 7.3 Printing Ink

Polystyrene powder (*M*_w_ 90 000, Sigma–Aldrich, USA) was dissolved in o‐xylene (*M*_w_ 106.17, Sigma–Aldrich, USA) at concentrations ranging from 0 to 20 mg/mL. The rheological properties of the polystyrene inks were measured using the MCR 102 rheometer (Anton Paar, Austria). Ink formulations for the study of different polymers (Figure 4c) were prepared as follows. SBS, PVP, and P3HT inks were prepared by dissolving SBS (*M_w_* 140 000, KTR 602, Kumho Petrochemical, Korea), PVP (*M_w_* 10 000, Sigma–Aldrich, USA), and P3HT (*M_w_* 50 000–100 000, Sigma–Aldrich, USA) in o‐xylene solvent, deionized water, and toluene (*M_w_* 92.14, Daejung, Korea), respectively at a concentration of 6 mg/mL. Polypyrrole ink (5 wt.%, dispersion in water, Sigma–Aldrich, USA) was used intact in the printing process.

### 7.4 Statistical Analysis

All geometrical parameters of fabricated wires (diameter and height) were measured from optical and SEM images using the open‐source software ImageJ (NIH, Bethesda, MD, USA). Reported values represent the mean ± standard deviation obtained from independent measurements. For dimensional analysis (Figure 1g), 60 nanowires were fabricated, and their diameter and height were measured. For diameter‐series analysis (Figure 4b), 10 independent fabrication cases were analyzed for each diameter condition. For polymer comparison experiments (Figure 4d), measurements were obtained from 10 independent fabrication cases per polymer. For molecular weight (M_w_) comparisons (Figure S10), 15 independent fabrication cases were analyzed for each M_w_. Rheological properties of the printing inks were obtained directly from the rheometer without additional statistical processing. Error bars in Figure 4b,d represent the standard deviation (SD) from the stated number of independent fabrication cases. Linear least‐squares fitting was applied to the laser power measurement data (Figure S11b), and the goodness of fit was evaluated using the coefficient of determination (R^2^). Statistical tests were not applied, as the measured data consist of geometrical dimensions and fabrication conditions.

## Author Contributions

J.P. conceived the idea. J.B. conducted experiments. C.Y. wrote the manuscript draft. C.Y. and J.B. analyzed experimental data and prepared visualizations. C.Y. and J.P. revised the manuscript. All authors commented on the results and final manuscript.

## Conflicts of Interest

The authors declare no conflicts of interest.

## Supporting information




**Supporting File 1**: smll74987‐sup‐0001‐SuppMat.docx.

**Supporting File 2**: smll74987‐sup‐0002‐MovieS1.mp4.

## Data Availability

The data that support the findings of this study are available from the corresponding author upon reasonable request.
