# Supplementary material for: Enabling Scalable Nanoscale 3D Printing via Laser‐Driven Termination
Source: Small. 2026 Aug 4;22(55):e14504. doi: 10.1002/smll.202514504 (PMC13630417; doi:10.1002/smll.202514504)
Supplement: Supplementary file 1 [file SMLL-22-e14504-s001.docx]

Supplementary Information

Enabling Scalable Nanoscale 3D Printing via Laser-Driven Termination

Chanbin Yoo^1,2,†^, Jongcheon Bae^1,3,†^, Jung Woo Lee^3*^, Ji-Tae Hong,^1,2^ Jun Chang Yang^1^, Seung Kwon Seol^1,2^, and Jaeyeon Pyo^1,2*^

^1^ Smart 3D Printing Research Team, Korea Electrotechnology Research Institute (KERI), Changwon 51543, Korea

^2^ Electric Energy & Materials Engineering, KERI School, University of Science and Technology (UST), Changwon 51543, Korea

^3^ School of Materials Science and Engineering, Pusan National University, Busan 46241, Korea

^†^ These authors have equally contributed to this work.

^*^ E-mail: jpyo@keri.re.kr (Jaeyeon Pyo), jungwoolee@pusan.ac.kr (Jung Woo Lee)

**Keywords**: 3D printing, nanostructure, polymer, laser ablation

**Figure S1.** **Various laser-based fabrication technologies.**

(1)	Lee, K. K. C.; Herman, P. R.; Shoa, T.; Haque, M.; Madden, J. D. W.; Yang, V. X. D. Microstructuring of Polypyrrole by Maskless Direct Femtosecond Laser Ablation. Advanced Materials 2012, 24 (9), 1243–1246. https://doi.org/10.1002/adma.201104170.

(2)	Yang, Q.; Hu, Z.; Seo, M.-H.; Xu, Y.; Yan, Y.; Hsu, Y.-H.; Berkovich, J.; Lee, K.; Liu, T.-L.; McDonald, S.; Nie, H.; Oh, H.; Wu, M.; Kim, J.-T.; Miller, S. A.; Jia, Y.; Butun, S.; Bai, W.; Guo, H.; Choi, J.; Banks, A.; Ray, W. Z.; Kozorovitskiy, Y.; Becker, M. L.; Pet, M. A.; MacEwan, M. R.; Chang, J.-K.; Wang, H.; Huang, Y.; Rogers, J. A. High-Speed, Scanned Laser Structuring of Multi-Layered Eco/Bioresorbable Materials for Advanced Electronic Systems. Nat Commun 2022, 13 (1), 6518. https://doi.org/10.1038/s41467-022-34173-0.

(3)	Xue, C.; Zhang, Y.; Li, L.; Hu, Y.; Chen, C.; Song, Y.; You, H.; Li, R.; Li, J.; Wu, D.; Chu, J. 3D Multiscale Micro-/Nanofolds by Femtosecond Laser Intermittent Ablation and Constrained Heating on a Shape Memory Polymer. ACS Appl. Mater. Interfaces 2021, 13 (19), 23210–23219. https://doi.org/10.1021/acsami.1c04049.

(4)	Srikanth, S.; Mohan, J. M.; Dudala, S.; Dubey, S. K.; Javed, A.; Goel, S. Direct UV Laser Writing System to Photolithographically Fabricate Optimal Microfluidic Geometries: Experimental Investigations. Materials Today: Proceedings 2020, 28, 799–803. https://doi.org/10.1016/j.matpr.2019.12.301.

(5)	Srikanth, S.; Dudala, S.; Raut, S.; Dubey, S. K.; Ishii, I.; Javed, A.; Goel, S. Optimization and Characterization of Direct UV Laser Writing System for Microscale Applications. J. Micromech. Microeng. 2020, 30 (9), 095003. https://doi.org/10.1088/1361-6439/ab92ea.

(6)	Paris, G.; Klinkusch, A.; Heidepriem, J.; Tsouka, A.; Zhang, J.; Mende, M.; Mattes, D. S.; Mager, D.; Riegler, H.; Eickelmann, S.; Loeffler, F. F. Laser-Induced Forward Transfer of Soft Material Nanolayers with Millisecond Pulses Shows Contact-Based Material Deposition. Applied Surface Science 2020, 508, 144973. https://doi.org/10.1016/j.apsusc.2019.144973.

(7)	Smits, E. C. P.; Walter, A.; De Leeuw, D. M.; Asadi, K. Laser Induced Forward Transfer of Graphene. Appl. Phys. Lett. 2017, 111 (17), 173101. https://doi.org/10.1063/1.5001712.

(8)	Eickelmann, S.; Tsouka, A.; Heidepriem, J.; Paris, G.; Zhang, J.; Molinari, V.; Mende, M.; Loeffler, F. F. A Low‐Cost Laser‐Based Nano‐3D Polymer Printer for Rapid Surface Patterning and Chemical Synthesis of Peptide and Glycan Microarrays. Adv Materials Technologies 2019, 4 (11), 1900503. https://doi.org/10.1002/admt.201900503.

(9)	Zheng, X.; Cheng, K.; Zhou, X.; Lin, J.; Jing, X. A Method for Positioning the Focal Spot Location of Two Photon Polymerization. AIP Advances 2017, 7 (9), 095318. https://doi.org/10.1063/1.4986102.

(10)	Zhang, W.; Wang, H.; Wang, H.; Chan, J. Y. E.; Liu, H.; Zhang, B.; Zhang, Y.-F.; Agarwal, K.; Yang, X.; Ranganath, A. S.; Low, H. Y.; Ge, Q.; Yang, J. K. W. Structural Multi-Colour Invisible Inks with Submicron 4D Printing of Shape Memory Polymers. Nat Commun 2021, 12 (1), 112. https://doi.org/10.1038/s41467-020-20300-2.

(11)	Zhu, J.; Zhang, Q.; Yang, T.; Liu, Y.; Liu, R. 3D Printing of Multi-Scalable Structures via High Penetration near-Infrared Photopolymerization. Nat Commun 2020, 11 (1), 3462. https://doi.org/10.1038/s41467-020-17251-z.

(12)	Skylar-Scott, M. A.; Gunasekaran, S.; Lewis, J. A. Laser-Assisted Direct Ink Writing of Planar and 3D Metal Architectures. Proc. Natl. Acad. Sci. U.S.A. 2016, 113 (22), 6137–6142. https://doi.org/10.1073/pnas.1525131113.

(13)	Ko, S. H.; Chung, J.; Pan, H.; Grigoropoulos, C. P.; Poulikakos, D. Fabrication of Multilayer Passive and Active Electric Components on Polymer Using Inkjet Printing and Low Temperature Laser Processing. Sensors and Actuators A: Physical 2007, 134 (1), 161–168. https://doi.org/10.1016/j.sna.2006.04.036.

(14)	Delrot, P.; Modestino, M. A.; Gallaire, F.; Psaltis, D.; Moser, C. Inkjet Printing of Viscous Monodisperse Microdroplets by Laser-Induced Flow Focusing. Phys. Rev. Applied 2016, 6 (2), 024003. https://doi.org/10.1103/PhysRevApplied.6.024003.

**Figure S2.** **3D nanowire printing via meniscus writing.** (**a**) Schematic images representing the procedure of meniscus writing for 3D nanowire fabrication. (**b**) Schematic and in-situ optical images of 3D nanowire printing. Scale bar: 10 μm.

Here is a detailed technical explanation of the meniscus writing principle, specifically focusing on the deposition process and its physical mechanisms. The process is initiated by the precise positioning of an ink-filled nozzle; upon approaching the substrate within several hundred nanometers, a femtoliter-scale liquid meniscus is established between the nozzle aperture and the substrate surface. Such a meniscus is not merely a liquid bridge but functions as a confined 3D reaction vessel. Notably, the diminutive scale of the ink meniscus results in a high surface-area-to-volume ratio, which significantly accelerates the solvent evaporation rate and promotes rapid phase transition into a solid state. Such ink meniscus is critical, as it dictates the initial resolution and provides the localized environment necessary for controlled phase transitions. The fundamental driving force for subsequent material deposition is evaporation-induced solidification. As the solvent undergoes evaporation at the air–liquid interface, the resulting local supersaturation triggers the spontaneous crystallization or self-assembly of the solute. To maintain steady-state deposition, a concurrent solution supply is driven from the nozzle reservoir to the meniscus base via capillary force, ensuring a continuous flux of solute to the growth front. During the deposition stage, the structural integrity and morphology of the architecture are governed by the kinetic balance between the evaporation rate ($v_{evaporation}$) and the printing velocity ($v$). When these rates are precisely matched ($v\approx v_{evaporation}$), the deposition follows the nozzle’s trajectory, enabling the fabrication of complex, freestanding architectures—such as vertical pillars and overhanging arches—without sacrificial supports. Following the completion of the desired structure, the process enters the termination phase, which has conventionally relied on the rapid retraction of nozzle. The rapid retraction technique involves a sudden, high-velocity retraction of the nozzle ($v\gg v_{deposition}$), causing the liquid meniscus to undergo mechanical rupture to achieve a separation between nozzle and printed structure.

**Figure S3. Repeatability.** (**a**) Repeated printing procedure. (**b**) Top view BF images of printed polymer nanostructures. Scale bars: 10 μm.

**Figure S4. Termination methods by different mechanisms.** Schematic illustrations and OM images representing the termination procedure for (**a**) conventional rapid retraction and (**b**) focused laser ablation. Scale bar: 10 μm.

**Figure S5. Rheological properties.** (**a**, **b**) Rheological properties of the photonic inks in Figure 2.

**Figure S6. Comparison of termination methods with long-duration printing processes.** Nanowires are fabricated by (**a**) rapid retraction at both the onset of printing and following extended durations and by (**b**) laser ablation at both the onset of printing and following extended durations. Scale bars: 10 μm.

**Figure S7. Height-dependent bending of tilted nanowires.** BF optical images of tilted 3D polymer nanostructure with different heights. Scale bars: 10 μm.

**Figure S8. SEM images of microcapillary nozzles.** Applicability to the nozzle tips having IDs of (**a**) 0.6 μm, (**b**) 1.0 μm, (**c**) 4.0 μm, and (**d**) 8.0 μm. Scale bars: 10 μm. Inset scale bar: 1 μm.

**Figure S9. Laser ablation on various substrate.** Applicability to substrate materials of (**a**) silicon, (**b**) PET, (**c**) copper, and (**d**) platinum substrates. Scale bars: 10 μm.

**Figure S10. Comparison of different molecular weight.** Laser ablation times were measured for polystyrene with molecular weights of (**a**) 35,000 and (**b**) 103,100. Laser process times in each molecular case. Scale bars: 10 μm.

**Figure S11.** **Laser power measurement.** (**a**) Spec sheet of laser diode. (**b**) Measured power intensity according to duty.

**Figure S12.** **Comparison of achievable aspect ratio of fabricated nanowires for different termination methods.** BF optical microscopy images of fabricated nanowires with increasing height until bending for (**a**) rapid retraction (PS: 1.6 mg/mL), (**b**) laser ablation (PS: 1.6 mg/mL), and (**c**) laser ablation (PS: 20 mg/mL). Scale bar: 30 μm.

At a same PS concentration (1.6 mg/mL), nanowires fabricated using termination by rapid

in (a) exhibited bending above an AR of 82.7, whereas termination by laser ablation in (b) enabled stable nanowires to be obtained at a higher AR of 104.5. This behavior is likely attributable to mechanical stress on the underlying printed wire during the separation of the nozzle from the printed structure in rapid retraction termination, leading to bending at a lower AR. In contrast, termination by laser ablation minimize the stress on printed structure, enabling in the higher aspect ratio (104.5). Under the same termination by laser ablation, increasing the PS concentration from 1.6 mg/mL in (b) to 20 mg/mL in (c) increased the achievable aspect ratio from 104.5 to 119.2. This result is possibly due to the higher polymer content within the meniscus, which facilitates the formation of a denser internal structure, resulting in wires with enhanced mechanical strength.

**Figure S13.** **Comparison of nanowire fabrication time depending on the PS concentration.** Bright-field (BF) optical images of nanowire fabrication using laser-ablation termination at PS concentrations of (**a**) 1.6 mg/ml and (**b**) 20.0 mg/ml. Scale bar: 3 μm.

At a PS concentration of 1.6 mg/mL, nanowire fabrication was performed at a printing speed of 1 μm/s, resulting in a total processing time of 52.5 s for a 50 μm-tall structure. In contrast, increasing the PS concentration to 20.0 mg/mL enabled stable nanowire fabrication at a printing speed of 1000 μm/s, reducing the total processing time to 3.7 s for the same structure height.

**Figure S14.** **Comparison of long-term throughput between rapid retraction and laser ablation.** (**a**) BF optical microscopy images of nanowires fabricated in series using termination by rapid retraction, showing the formation of defective structures beyond 60 printed nanowires. (**b**) BF optical microscopy image showing defect formation resulting from a progressive increase in polymer concentration in the capillary nozzle caused by solvent evaporation. (**c**) BF optical microscopy images of nanowires fabricated in series using termination by laser ablation, demonstrating stable fabrication of more than 140 nanowires without defects. Scale bar: 10 μm.

**Figure S15.** **Nanowires fabricated at different PS concentrations.** (**a, b**) SEM images of nanowires fabricated at PS concentrations of 1.6 mg/mL (**a**) and 0.2 mg/mL (**b**). Insets highlight the printed diameter of nanowires. Scale bars: 10 μm. Inset scale bar: 1 μm.

As the PS concentration decreases, the nanowire diameter decreases due to reduced polymer content in the meniscus during nanowire formation.

**Figure S16.** **Fabrication of a 3D house structure.** Scale bars: 10 μm.

**Figure S17.** **Various 3D structures via termination by laser ablation.** (**a**) branched nanostructure. (**b**) nanowire-aligned grating. Scale bars: 10 μm.

**Figure S18. Laser termination demonstrated for diverse polymer systems.** (**a**) Polyvinylidene fluoride (PVDF) and (**b**) Hydroxypropyl cellulose (HPC). Scale bars: 10 µm.

**Figure S19. Laser-driven termination-based fabrication of PS matrix nanowires embedded with quantum dots.** (**a**) Bright-field optical images showing nanowire fabrication using a Cd-based quantum dot (QD)-integrated polystyrene ink. (**b**) Magnified dark-field image of the Cd-based QD nanowire region highlighted in (a). (**c**) Top-view optical microscopy image of the Cd-based QD nanowire. (**d**) Top-view PL image of the Cd-based QD nanowire. (**e**) Bright-field optical images showing nanowire fabrication using a perovskite quantum dot (PeQD)-integrated polystyrene ink. (**f**) Magnified dark-field image of the PeQD nanowire region highlighted in (e). (**g**) Top-view optical microscopy image of the PeQD nanowire. (**h**) Top-view PL image of the PeQD nanowire. Scale bars: 10 µm. (a,e) Scale bars: 5 µm. (b-d, f-h)

**Figure S20. Various applications of meniscus writing.**

(1)	Kim, J. H.; Lee, S.; Wajahat, M.; Jeong, H.; Chang, W. S.; Jeong, H. J.; Yang, J.-R.; Kim, J. T.; Seol, S. K. Three-Dimensional Printing of Highly Conductive Carbon Nanotube Microarchitectures with Fluid Ink. *ACS Nano* **2016**, *10* (9), 8879–8887. https://doi.org/10.1021/acsnano.6b04771.

(2)	Lee, S.; Wajahat, M.; Kim, J. H.; Pyo, J.; Chang, W. S.; Cho, S. H.; Kim, J. T.; Seol, S. K. Electroless Deposition-Assisted 3D Printing of Micro Circuitries for Structural Electronics. *ACS Appl. Mater. Interfaces* **2019**, *11* (7), 7123–7130. https://doi.org/10.1021/acsami.8b18199.

(3)	Kim, J. H.; Chang, W. S.; Kim, D.; Cho, S. H.; Seol, S. K. Conductivity Enhancement of Stretchable PEDOT:PSS Nanowire Interconnect Fabricated by Fountain-Pen Lithography. *Materials Chemistry and Physics* **2014**, *147* (3), 1171–1174. https://doi.org/10.1016/j.matchemphys.2014.06.075.

(4)	Kim, J. H.; Chang, W. S.; Kim, D.; Yang, J. R.; Han, J. T.; Lee, G.; Kim, J. T.; Seol, S. K. 3D Printing of Reduced Graphene Oxide Nanowires. *Advanced Materials* **2015**, *27* (1), 157–161. https://doi.org/10.1002/adma.201404380.

(5)	Chang, W. S.; Kim, J. H.; Kim, D.; Cho, S. H.; Kwon Seol, S. Individually Addressable Suspended Conducting‐Polymer Wires in a Chemiresistive Gas Sensor. *Macro Chemistry & Physics* **2014**, *215* (17), 1633–1638. https://doi.org/10.1002/macp.201400220.

(6)	Liu, Y.; Lee, K.; Liu, H.; Li, C.; Zeng, X.; Liu, R.; Chen, Y.; Chen, Z.; Yang, J.; Huan, X.; Park, I.; Kim, J. T.; Cheng, X. Universal 3D‐Printing of Suspended Metal Oxide Nanowire Arrays on MEMS for AI‐Optimized Combinatorial Gas Fingerprinting. *Advanced Science* **2025**, e11794. https://doi.org/10.1002/advs.202511794.

(7)	Sim, H. H.; Kim, J. H.; Bae, J.; Yoo, C.; Kim, D.; Pyo, J.; Seol, S. K. 3D‐Printing of Freestanding Pure MXene Microarchitectures. *Small* **2025**, *21* (20), 2409198. https://doi.org/10.1002/smll.202409198.

(8)	Kim, J. T.; Pyo, J.; Rho, J.; Ahn, J.-H.; Je, J. H.; Margaritondo, G. Three-Dimensional Writing of Highly Stretchable Organic Nanowires. *ACS Macro Lett.* **2012**, *1* (3), 375–379. https://doi.org/10.1021/mz200249c.

(9)	Lee, H.; Wang, Z.; Rao, Q.; Lee, S.; Huan, X.; Liu, Y.; Yang, J.; Chen, M.; Ki, D.; Kim, J. T. Additive Manufacturing of Thermoelectric Microdevices for 4D Thermometry. *Advanced Materials* **2023**, *35* (35), 2301704. https://doi.org/10.1002/adma.202301704.

(10)	Chen, M.; Zhou, Z.; Hu, S.; Huang, N.; Lee, H.; Liu, Y.; Yang, J.; Huan, X.; Xu, Z.; Cao, S.; Cheng, X.; Wang, T.; Yu, S. F.; Chan, B. P.; Tang, J.; Feng, S.; Kim, J. T. 3D Printing of Arbitrary Perovskite Nanowire Heterostructures. *Adv Funct Materials* **2023**, *33* (15), 2212146. https://doi.org/10.1002/adfm.202212146.

(11)	Chen, M.; Hu, S.; Zhou, Z.; Huang, N.; Lee, S.; Zhang, Y.; Cheng, R.; Yang, J.; Xu, Z.; Liu, Y.; Lee, H.; Huan, X.; Feng, S.-P.; Shum, H. C.; Chan, B. P.; Seol, S. K.; Pyo, J.; Tae Kim, J. Three-Dimensional Perovskite Nanopixels for Ultrahigh-Resolution Color Displays and Multilevel Anticounterfeiting. *Nano Lett.* **2021**, *21* (12), 5186–5194. https://doi.org/10.1021/acs.nanolett.1c01261.

(12)	Bae, J.; Yoo, C.; Kim, S.; Ahn, J.; Sim, H. H.; Kim, J. H.; Kim, J. H.; Yoon, S.-Y.; Kim, J. T.; Seol, S. K.; Pyo, J. Three-Dimensional Printing of Structural Color Using a Femtoliter Meniscus. *ACS Nano* **2023**, *17* (14), 13584–13593. https://doi.org/10.1021/acsnano.3c02236.

(13)	Bae, J.; Lee, J. W.; Hong, J.; Yang, J. C.; Seol, S. K.; Pyo, J. 3D Printing of Multi‐Layer Diffraction Gratings for Additive Mixing of Structural Colors. *Small* **2026**, *22* (22), e14857. https://doi.org/10.1002/smll.202514857.

(14)	Bae, J.; Kim, S.; Ahn, J.; Sim, H. H.; Wajahat, M.; Kim, J. H.; Yoon, S.-Y.; Kim, J. T.; Seol, S. K.; Pyo, J. Nanoscale 3D Printing of Quantum Dots on Paper. *Adv Eng Mater* **2021**, *23* (9), 2100339. https://doi.org/10.1002/adem.202100339.

(15)	Bae, J.; Lee, S.; Ahn, J.; Kim, J. H.; Wajahat, M.; Chang, W. S.; Yoon, S.-Y.; Kim, J. T.; Seol, S. K.; Pyo, J. 3D-Printed Quantum Dot Nanopixels. *ACS Nano* **2020**, *14* (9), 10993–11001. https://doi.org/10.1021/acsnano.0c04075.

(16)	Bae, J.; Yoo, C.; Yoon, S.-Y.; Seol, S. K.; Pyo, J. Emission Directionality of 3D-Printed Photonic Nanowires. *ACS Nano* **2024**, *18* (25), 16265–16273. https://doi.org/10.1021/acsnano.4c02820.

(17)	Kim, J. H.; Park, S.; Ahn, J.; Pyo, J.; Kim, H.; Kim, N.; Jung, I. D.; Seol, S. K. Meniscus‐Guided Micro‐Printing of Prussian Blue for Smart Electrochromic Display. *Advanced Science* **2023**, *10* (3), 2205588. https://doi.org/10.1002/advs.202205588.

(18)	Chen, M.; Yang, J.; Wang, Z.; Xu, Z.; Lee, H.; Lee, H.; Zhou, Z.; Feng, S.; Lee, S.; Pyo, J.; Seol, S. K.; Ki, D.; Kim, J. T. 3D Nanoprinting of Perovskites. *Advanced Materials* **2019**, *31* (44), 1904073. https://doi.org/10.1002/adma.201904073.

(19)	Cao, S.; Liu, Y.; Gan, Z.; Hu, S.; Yang, J.; Wang, Z.; Yuan, M.; Li, W.; Kim, J. T. Freeform Fabrication of Layered Halide Perovskite Nanowire Heterojunctions. *Advanced Materials* **2026**, *38* (27), e22768. https://doi.org/10.1002/adma.202522768.

(20)	Seol, S. K.; Chang, W. S.; Kim, D.; Jung, S. Carbon Nanotube-Conducting Polymer Composite Wires Formed by Fountain Pen Growth (FPG) Route. *RSC Adv.* **2012**, *2* (24), 8926. https://doi.org/10.1039/c2ra21076b.

(21)	Yoo, J.; Pyo, J.; Je, J. H. Single Inorganic–Organic Hybrid Nanowires with Ambipolar Photoresponse. *Nanoscale* **2014**, *6* (7), 3557–3560. https://doi.org/10.1039/C3NR05910C.

(22)	Kim, J. T.; Seol, S. K.; Pyo, J.; Lee, J. S.; Je, J. H.; Margaritondo, G. Three-Dimensional Writing of Conducting Polymer Nanowire Arrays by Meniscus-Guided Polymerization. *Advanced Materials* **2011**, *23* (17), 1968–1970. https://doi.org/10.1002/adma.201004528.

(23)	Lee, J.; Oh, S.; Pyo, J.; Kim, J.-M.; Je, J. H. A Light-Driven Supramolecular Nanowire Actuator. *Nanoscale* **2015**, *7* (15), 6457–6461. https://doi.org/10.1039/C5NR01118C.

(24)	Kim, W.-G.; Kim, S.-J.; Lee, I. H.; Devaraj, V.; Jeon, N.; Park, C.; Kim, M.; Lee, D.; Jeon, I.; Lee, J.-M.; Tae Kim, J.; Rho, J.; Oh, J.-W. Nanofountain Pen for Writing Hybrid Plasmonic Architectures. *Small Structures* **2024**, *5* (1), 2300260. https://doi.org/10.1002/sstr.202300260.

(25)	Huan, X.; Lee, S.; Lee, H.; Xu, Z.; Yang, J.; Chen, M.; Liu, Y.; Kim, J. T. One-Step, Continuous Three-Dimensional Printing of Multi-Stimuli-Responsive Bilayer Microactuators via a Double-Barreled Theta Pipette. *ACS Appl. Mater. Interfaces* **2021**, *13* (36), 43396–43403. https://doi.org/10.1021/acsami.1c12574.

(26)	Yang, J.; Huan, X.; Liu, Y.; Lee, H.; Chen, M.; Hu, S.; Cao, S.; Kim, J. T. Three-Dimensional Printing of Dipeptides with Spatioselective Programming of Crystallinity for Multilevel Anticounterfeiting. *Nano Lett.* **2022**, *22* (19), 7776–7783. https://doi.org/10.1021/acs.nanolett.2c01761.

(27)	Kim, W.-G.; Lee, J.-M.; Yang, Y.; Kim, H.; Devaraj, V.; Kim, M.; Jeong, H.; Choi, E.-J.; Yang, J.; Jang, Y.; Badloe, T.; Lee, D.; Rho, J.; Kim, J. T.; Oh, J.-W. Three-Dimensional Plasmonic Nanocluster-Driven Light–Matter Interaction for Photoluminescence Enhancement and Picomolar-Level Biosensing. *Nano Lett.* **2022**, *22* (12), 4702–4711. https://doi.org/10.1021/acs.nanolett.2c00790.

(28)	Yang, U.; Kang, B.; Yong, M.; Yang, D.; Choi, S.; Je, J. H.; Oh, S. S. Type‐Independent 3D Writing and Nano‐Patterning of Confined Biopolymers. *Advanced Science* **2023**, *10* (13), 2207403. https://doi.org/10.1002/advs.202207403.

(29)	Wajahat, M.; Lee, S.; Kim, J. H.; Chang, W. S.; Pyo, J.; Cho, S. H.; Seol, S. K. Flexible Strain Sensors Fabricated by Meniscus-Guided Printing of Carbon Nanotube–Polymer Composites. *ACS Appl. Mater. Interfaces* **2018**, *10* (23), 19999–20005. https://doi.org/10.1021/acsami.8b04073.

(30)	Yang, J.; Chen, M.; Lee, H.; Xu, Z.; Zhou, Z.; Feng, S.-P.; Kim, J. T. Three-Dimensional Printing of Self-Assembled Dipeptides. *ACS Appl. Mater. Interfaces* **2021**, *13* (17), 20573–20580. https://doi.org/10.1021/acsami.1c03062.

(31)	Pyo, J.; Kim, J. T.; Lee, J.; Yoo, J.; Je, J. H. 3D Printed Nanophotonic Waveguides. *Advanced Optical Materials* **2016**, *4* (8), 1190–1195. https://doi.org/10.1002/adom.201600220.

**Figure S21. Comparison of representative direct writing technologies for the fabrication of freestanding micro- and nanowires.**

(1)	Lejeune, M.; Chartier, T.; Dossou-Yovo, C.; Noguera, R. Ink-Jet Printing of Ceramic Micro-Pillar Arrays. Journal of the European Ceramic Society 2009, 29 (5), 905–911. https://doi.org/10.1016/j.jeurceramsoc.2008.07.040.

(2)	Ko, S. H.; Chung, J.; Hotz, N.; Nam, K. H.; Grigoropoulos, C. P. Metal Nanoparticle Direct Inkjet Printing for Low-Temperature 3D Micro Metal Structure Fabrication. J. Micromech. Microeng. 2010, 20 (12), 125010. https://doi.org/10.1088/0960-1317/20/12/125010.

(3)	Galliker, P.; Schneider, J.; Eghlidi, H.; Kress, S.; Sandoghdar, V.; Poulikakos, D. Direct Printing of Nanostructures by Electrostatic Autofocussing of Ink Nanodroplets. Nat Commun 2012, 3 (1), 890. https://doi.org/10.1038/ncomms1891.

(4)	An, B. W.; Kim, K.; Lee, H.; Kim, S.; Shim, Y.; Lee, D.; Song, J. Y.; Park, J. High‐Resolution Printing of 3D Structures Using an Electrohydrodynamic Inkjet with Multiple Functional Inks. Advanced Materials 2015, 27 (29), 4322–4328. https://doi.org/10.1002/adma.201502092.

(5)	Reiser, A.; Lindén, M.; Rohner, P.; Marchand, A.; Galinski, H.; Sologubenko, A. S.; Wheeler, J. M.; Zenobi, R.; Poulikakos, D.; Spolenak, R. Multi-Metal Electrohydrodynamic Redox 3D Printing at the Submicron Scale. Nat Commun 2019, 10 (1), 1853. https://doi.org/10.1038/s41467-019-09827-1.

(6)	Hu, S.; Wang, T.; Zhou, Z.; Liu, Y.; Yang, J.; Zou, T.; Li, X.; Jiang, T.; Lee, H.; Huan, X.; Park, C.; Jeon, N.; Feng, S.-P.; Kim, G.-Y.; Choi, S.-Y.; Noh, Y.-Y.; Yuan, M.; Chen, M.; Rho, J.; Yu, S. F.; Kim, J. T. Nanoprinting with Crystal Engineering for Perovskite Lasers. ACS Nano 2025, 19 (50), 42648–42657. https://doi.org/10.1021/acsnano.5c16906.

(7)	Takada, K.; Wu, D.; Chen, Q.-D.; Shoji, S.; Xia, H.; Kawata, S.; Sun, H.-B. Size-Dependent Behaviors of Femtosecond Laser-Prototyped Polymer Micronanowires. Opt. Lett. 2009, 34 (5), 566. https://doi.org/10.1364/OL.34.000566.

(8)	Ushiba, S.; Masui, K.; Taguchi, N.; Hamano, T.; Kawata, S.; Shoji, S. Size Dependent Nanomechanics of Coil Spring Shaped Polymer Nanowires. Sci Rep 2015, 5 (1), 17152. https://doi.org/10.1038/srep17152.

(9)	Purtov, J.; Verch, A.; Rogin, P.; Hensel, R. Improved Development Procedure to Enhance the Stability of Microstructures Created by Two-Photon Polymerization. Microelectronic Engineering 2018, 194, 45–50. https://doi.org/10.1016/j.mee.2018.03.009.

(10)	Nan, B.; Galindo-Rosales, F. J.; Ferreira, J. M. F. 3D Printing Vertically: Direct Ink Writing Free-Standing Pillar Arrays. Materials Today 2020, 35, 16–24. https://doi.org/10.1016/j.mattod.2020.01.003.

(11)	Kim, F.; Yang, S. E.; Ju, H.; Choo, S.; Lee, J.; Kim, G.; Jung, S.; Kim, S.; Cha, C.; Kim, K. T.; Ahn, S.; Chae, H. G.; Son, J. S. Direct Ink Writing of Three-Dimensional Thermoelectric Microarchitectures. Nat Electron 2021, 4 (8), 579–587. https://doi.org/10.1038/s41928-021-00622-9.

(12)	Bae, J.; Lee, S.; Ahn, J.; Kim, J. H.; Wajahat, M.; Chang, W. S.; Yoon, S.-Y.; Kim, J. T.; Seol, S. K.; Pyo, J. 3D-Printed Quantum Dot Nanopixels. ACS Nano 2020, 14 (9), 10993–11001. https://doi.org/10.1021/acsnano.0c04075.

(13)	Bae, J.; Kim, S.; Ahn, J.; Sim, H. H.; Wajahat, M.; Kim, J. H.; Yoon, S.-Y.; Kim, J. T.; Seol, S. K.; Pyo, J. Nanoscale 3D Printing of Quantum Dots on Paper. Adv Eng Mater 2021, 23 (9), 2100339. https://doi.org/10.1002/adem.202100339.

(14)	Bae, J.; Yoo, C.; Yoon, S.-Y.; Seol, S. K.; Pyo, J. Emission Directionality of 3D-Printed Photonic Nanowires. ACS Nano 2024, 18 (25), 16265–16273. https://doi.org/10.1021/acsnano.4c02820.
